# Clinical Evidence of Low-Carbohydrate Diets against Obesity and Diabetes Mellitus

**DOI:** 10.3390/metabo13020240

**Published:** 2023-02-06

**Authors:** Eleni Pavlidou, Sousana K. Papadopoulou, Aristeidis Fasoulas, Maria Mantzorou, Constantinos Giaginis

**Affiliations:** 1 Department of Food Science and Nutrition, School of Environment, University of the Aegean, 81400 Myrina, Greece; 2 Department of Nutritional Sciences and Dietetics, Faculty of Health Sciences, International Hellenic University, 57001 Thessaloniki, Greece

**Keywords:** obesity, low-carbohydrate diet, diabetes mellitus, healthy diet, weight control, glucose

## Abstract

The popularity of low-carbohydrate diets (LCDs) in the last few decades has motivated several research studies on their role in a variety of metabolic and non-morbid conditions. The available data of the results of these studies are put under the research perspective of the present literature review of clinical studies in search of the effects of LCDs on Obesity and Diabetes Mellitus. The electronic literature search was performed in the databases PubMed, Cochrane, and Embase. The literature search found seven studies that met the review’s inclusion and exclusion criteria out of a total of 2637 studies. The included studies involved randomized controlled trials of at least 12 weeks’ duration, in subjects with BMI ≥ 25 kg/m^2^, with dietary interventions. The results of the study on the effects of LCDs on obesity showed their effectiveness in reducing Body Mass Index and total body fat mass. In addition, LCDs appear to cause drops in blood pressure, low-density lipoprotein (LDL), and triglycerides, and seem to improve high-density lipoprotein (HDL) values. Regarding the effectiveness of LCDs in Diabetes Mellitus, their effect on reducing insulin resistance and fasting blood glucose and HbA1c values are supported. In conclusion, the results suggest the critical role of LCDs to improve the health of people affected by obesity or diabetes.

## 1. Introduction

The necessity for creating efficient treatment alternatives for those who are affected by obesity or diabetes has been increasing [1]. The advantages of structured lifestyle therapies have been reported through numerous surveys in a large range of people categories, such as hyperglycaemia that was not dependent on diabetes [2], cardiovascular disease [3], and type 2 diabetes [4]. However, it is crucial to note that only a change in lifestyle can allow a person to successfully lose weight significantly and sustainably over time [5]. Recently, some medical professionals and professional organizations have endorsed Low-Carb Diets (LCDs) as a legitimate and successful choice for treating diabetes and obesity [6]. It is a scientific area, though, and as such it is rife with controversies, contradictory findings, differing expert perspectives, and potential ambiguity for both healthcare professionals and their patients. This approach to treat diabetes and obesity is not novel [7].

In its 2002 report, the Institute of Medicine (IOM) established an RDA for carbohydrates of 130 g/d for adults and children aged ≥1 year. This value is based on the amount ofsugars and starches required to provide the brain with an adequate supply of glucose. The IOM set an acceptable macronutrient distribution range (AMDR) for carbohydrates of 45–65% of total calories. According to the level of carbohydrate restriction used in contemporary clinical practice, less than 20 g/day, or 10% of overall calorie consumption, isconsidered an especially small carbohydrate consumption (although some suggest a maximum of 50 g). Fewer than 30 g/day, or less than 26% of overall energy intake from carbohydrates, is generally considered as the cutoff for “low” carbohydrate consumption. A carbohydrate consumption beyond 50 g per day is typically insufficient for ketogenesis, because in general, the more carbohydrate restriction, the greater the degree of ketogenesis [8]. As a result, “low carb” and “ketogenic” are not nutritional terms that are interchangeable but rather overlap each other. The limitation of carbohydrates is currently accepted as a valid therapeutic approach in international guidelines for the nutritional management of type 2 diabetes [9].

LCDs were found to be favorable for additional body mass, lipids, such as High-Density Lipoprotein (HDL), cholesterol, and triglycerides, whereas Low-Density Lipoprotein (LDL) and glucose metabolism were not affected, according to clinical trials that lasted up to two years [10]. These results coincide with a period of transition in the nutritional epidemiology dogma about the risks of dietary fat. Substantial studies have currently supported evidence that the impact of overall fat consumption on health is not significant, while diverse forms of fat should be examined [11], in contrast to previous nutritional recommendations that diet is healthier when fat content decreases. There are pharmacological treatments available [12], but just like with lifestyle modifications, there are a variety of results that may be anticipated from the different medications concerning the amount of body mass lowering and the improvements displayed in glycemic control [13].

BMI ≥ 30 kg/m^2^ is related to a high probability of developing type 2 diabetes, cardiovascular disease, cancer, and death [14]. The improvement in patient’s overall health is the main goal of treating obesity rather than only reducing the patient’s body mass [15]. However, no particular treatment for obesity has been shown in clinical trials to prolong life, and unintended weight loss may potentially be linked to a higher mortality rate [16].

Clinical evidence tracking the impact of lowering the body mass, whether voluntary or not, has shown conflicting results [17]. However, it is hoped that losing weight will lower the burden of disease and mortality linked to obesity. As a result, a moderate and gradual weight loss, often between 5% and 10% of starting weight, is recommended [18]. On the other hand, those who are more vulnerable to metabolic and cardiovascular problems [19] might profit by establishing more ambitious goals for themselves. The first-line treatment for weight loss involves dietary changes, as well as an increase in physical activity and a decrease in the amount of time spent on sedentary activities like sitting. Changes to one’s caloric intake, eating routine, and nutrient composition are a few examples of these alterations. A diet that meets the requirements of being risk-free, nourishing, nutritionally sufficient, culturally acceptable, economically feasible, and effective over the long term is considered as ideal [20]. It ought to guarantee that diet is consistently followed. The bulk of recommendations urge a daily calorie deficit of 600 kcal and a decrease in fat intake [21]. In this aspect, the “Mediterranean Diet” is frequently regarded as the best strategy since long-term epidemiological data demonstrate reduced overall morbidity and mortality rates in people that follow such a regimen [22]. This is ascribed to the fact that the “Mediterranean Diet” emphasizes on consuming more fruits, vegetables and unprocessed carbs, while decreasing the intake of fat, particularly saturated fat, and refined sugar. 

LCDs have recently received attention from various scientific organizations due to their applicability and efficacy in treating obesity [23]. LCDs are a broad category that covers several different methods of carbohydrate restriction [24]. In modern LCDs, proteins and not lipids are often the main source of energy. LCDs retain muscle mass, while reducing the harmful effects of aberrant lipid metabolism, which commonly necessitates the usage of high-cost protein supplements [25]. In people affected by obesity, an increased probability of kidney problems and ingestion due to an excessive amount of protein through their diet may be harmful for their kidney health [26]. This is due to the possibility that increased daily protein consumption may be related to a more accelerated reduction in glomerular filtration over time. Even though this is a contentious subject, there are some data that suggest that a larger daily protein intake may be connected to a more accelerated reduction in glomerular filtration over time. 

Besides all the benefits of LCDs, the body experiences stress when its intake of carbohydrates is limited because it must find alternative sources of energy. The “keto flu,” or adverse effects from a low-carb diet, might include headaches and nausea. If not addressed, the shortage of carbs will also result in hormonal shifts, fluid and mineral loss, and fluid and mineral retention [27].

## 2. Materials and Methods

This is a comprehensive review. In this regard, a thorough search of the PubMed, Cochrane, and Embase databases was conducted to collect all randomized human clinical trials written in English up to 1 October 2022. References of published studies were also carefully searched to further explore the currently available literature. The studies included had to meet the following criteria: a randomized controlled clinical trial; at least 12 weeks duration of dietary intervention; and reports for body mass or BMI of all intervention groups.

### 2.1. Data Collection

Four authors independently reviewed titles and abstracts, and relevant papers were retrieved in full-text format. Results given in existing papers and supplements served as the main resource of knowledge for all published trials. Four of the writers used the Cochrane Collaboration’s [28] characteristics to assess the risk of bias; disputes were settled; and reported bias was evaluated for each primary outcome.

### 2.2. Selection Criteria

Τhe inclusion requirements for this review were the following: (1) randomized controlled trial design; (2) adult participants (at least 18 years old); (3) BMI ≥ 25 kg/m^2^, containing only obese individuals; (4) the duration of the intervention lasted at least one month [29] and up to two years [30]; (5) the research was published during the last ten years, 2012–2022. Weight loss was included as an outcome result, and an LCD was also considered as a dietary intervention. A given consumption of carbohydrates ≤40% of overall energy consumption or a specific mention of the Atkins diet, with a consumption of only 20–40 g of carbohydrates per day in the initial stage, or a carbohydrate consumption ≤20% of total energy intake, were considered as carbohydrate-restricted diets [31]. When alternative procedures like medication or surgery were mentioned, the studies were excluded. Systematic reviews and meta-analysis were excluded as well as studies that did not examine a typically LCD.

## 3. Results

**Figure 1:**
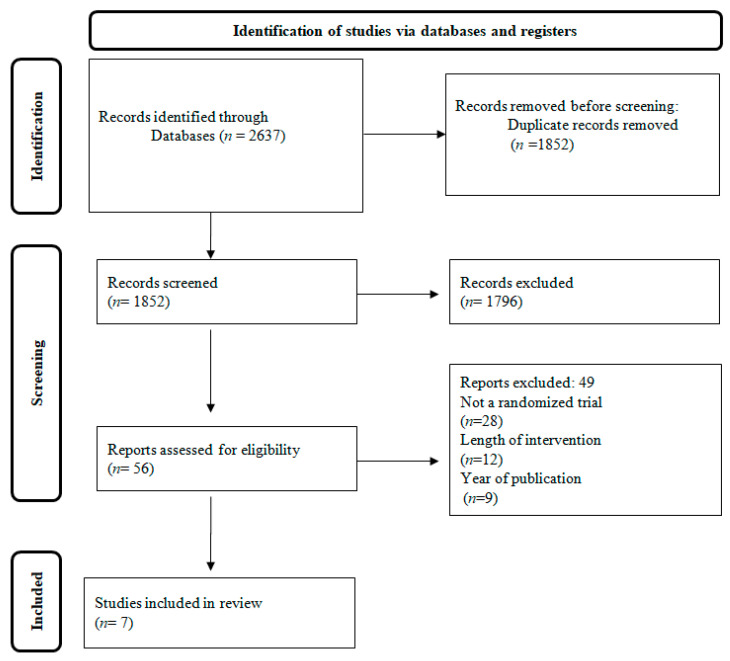
Flow chart of the study population.

Figure 1 depicts the research screening process in the form of a flowchart. At first, 2637 studies were retrieved. Using the aforementioned criteria we excluded 1852 articles, and 49 out of the 56 studies that were assessed were further excluded after reading the entire text. In the end, 7 papers fulfilled all the insertion criteria and were chosen for the current evaluation.

### 3.1. Characteristics of the Study

The basic characteristics of the seven randomized controlled trials included in this review are shown in Table 1. The total of participants was 1394. The measured parameters were body weight, LDL, HDL, triglycerides, cholesterol, apolipoprotein, B: A1 ratios, BMI, body fat, and insulin secretion.

### 3.2. Results of the Diets

In the study by Jenkins et al. [30] saturated fat intake was comparable across all treatment groups; however, mono-unsaturated fatty acids (MUFAs), vegetable proteins, and soy protein intake were considerably increased in the LCD intervention group, where available carbohydrate consumption was considerably reduced. The LCD attrition rate was 50% (10/20), while the high carbohydrate diet attrition rate was 32% (6/19), resulting in an overall attrition rate of 41% (16/39). There was no difference in the number of individuals that did not carry out the survey (containing dropouts and withdrawals) among regimens. According to the mean macronutrient consumption values in the study by Guldbrand et al. [30], compliance with the planned nutrition was excellent in both groups for the first six months. The macronutrient composition of the low-fat group did not change considerably throughout the study, whereas the LCD group’s energy from fat increased. Despite a reduction in total energy intake, the group that had fewer carbohydrates did not show a reduction in total fat intake, whereas the group that consumed fewer fats did. The diet records showed that during the period of trial, there was no change in the number of calories consumed by each group; however, the proportion of total energy consumed that was made up by saturated fat increased in the group that consumed fewer carbohydrates.

#### 3.2.1. Weight Loss, BMI, Lipids

LCDs helped study participants to lose more weight, overall, throughout the course of the trial, with a considerable difference among groups: the body mass lowering on the LCD, being 6.9 kg (95% CI 7.7 to 6.1) and 5.8 kg (95% CI 6.6 to 5.1), and on the control diet (therapy variation (95% CI) 1.1 kg (2.1 to 0.0); *p* = 0.047). This was contrasted with the 5.8 kg (95% CI 6.6 to 5.1) weight loss achieved on the control diet. Following an LCD resulted in a similar overall decrease in BMI compared to a High Carb Diet (HCD) (therapy variation, 95% confidence interval: 0.4 kg/m^2^ (0.8 to 0.0); *p* = 0.039). For both body mass and BMI treatments, the proportion of participants who completed the trial varied significantly (treatment difference (95% CI): 1.8 kg (3.1 to 0.6); *p* = 0.004 and 0.7 kg/m^2^ (1.1 to 0.2); *p* = 0.004, respectively) [32].

In contrast to those in the LCD group, those on the HCD increased their reported exercise (treatment variation (95% CI) 9.3 (16.4 to 2.2) METs; *p* = 0.012). Although this was the case, the HCD group did not lose more weight. In terms of body fat percentage, waist circumference, or feelings of satiety, no differences between treatment groups were noted [32]. Percent decreases in body mass and BMI compared to the control group were 7.9%, 3.8% and 7.8%, 3.9% with the decreased-carbohydrate diet, respectively, and 6.7%, 4.4%, and 6.8%, 4.5% with the low-fat diet (*p* = 0.001 compared to control for both groups; *p* = not significant between interventions). Both dietary regimens reduced energy intake in a comparable way. Fat consumption was lowered in the reduced fat group, whereas carbohydrate and protein consumption remained similar.

In combination with the considerable reduction in carbohydrate consumption, a slight rise in protein and a reduction in fat consumption was noticed in the reduced-carbohydrate group. The amount of sucrose, saturated fatty acids, and n-3 and n-6 polyunsaturated fatty acids (percent of overall calorie intake) was similar at baseline but significantly differed among participants at 6 months [33]. The mean 1st-year body mass alteration for the healthy low-fat nutrition participants was 5.3 kg (95% CI, 5.9 kg to 4.7 kg) and 6.0 kg (95% CI, 6.6 kg to 5.4 kg) for the LCD group of healthy individuals, though without reaching statistical significance. Within each group, there was the same variety of body mass alteration of roughly 4 kg (±4.1 kg to ±4.2 kg) [34]. BMI, body mass, fat mass, waist circumference, waist-to-hip fraction, and systolic blood pressure were all reduced with the HP (High protein) and S (No carbs) diets after 3, 6, and 9 months. HP diet resulted in a greater drop on BMI (1.3 kg/m^2^ vs. 1.2 kg/m^2^: *p* = 0.05), body mass (4.2 kg vs. 4.1 kg: *p* = 0.05), fat mass (4.1 kg vs. 4.2 kg: *p* = 0.05), and systolic blood pressure (7.1 mmHg vs. 2.1). At 3, 6, and 9 months there were no significant differences on waist-to-hip ratio or diastolic blood pressure [35]. At the end of the trial [32] the decrease in the LCD was higher for LDL-C (treatment variation (95% CI) 0.49 mmol/L (0.70 to 0.28); *p* = 0.001), for total cholesterol (TC) (0.62 mmol/L (0.86 to 0.37); *p* = 0.001, for TC:HDL-C 0.57 (0.83 to 0.32); *p* = 0.001), and for LDL-C:HDL-C 0.42. No treatment alteration concerning HDL-C was noted. In the participants that completed the trial the same tendency was noted. The intervention variation was arithmetically greater for LDL-C (0.60 mmol/L (0.84 to 0.36); *p* = 0.0001), TC (0.73 mmol/L (1.00 to 0.45); *p* = 0.0001), TC:HDL-C (0.68 (0.97 to 0.39); *p* = 0.0001), and LDL-C:HDL-C (0.53 (0.73 to 0.32); *p* = 0.0001). LDL-C and the TC:HDL-C fraction were continuously reduced in LCD individuals throughout the research, but HDL-C remained unaltered compared to baseline [32].

In addition, the study by Haufe et al. highlights the fact that after implementing a six-month low-carbohydrate or low-fat diet, in addition to body weight loss, a significant improvement in left ventricular mass was observed in overweight and obese individuals [33].

#### 3.2.2. HbA1c, Blood Glucose, Serum Insulin, Insulin Resistance, Apolipoproteins, and Blood Pressure

Some other advantages of following a LCD, besides weight loss, were also noted. More specifically, at the end of one trial [32] the LCD group had lower levels of ApoB and ApoB:ApoA1 fraction than the HCD group (treatment difference (95% CI) 0.11 g/L (0.16 to 0.06; *p* = 0.001 and 0.05 (0.09 to 0.02; *p* = 0.003, respectively). However, the levels of apoA1 did not significantly differ across diets. Apolipoprotein changes in the participants that completed the trial followed the same trend as the alterations in the entire group. LCD decreased ApoB and ApoB:ApoA1 fractions during the trial in comparison to baseline [32]. The reduction in hs-CRP was the same for all regimens. HbA1c, fasting blood glucose, insulin, and insulin tolerance (as measured by HOMA protocol) were decreased in the same way in all regimens during the trial. Systolic and diastolic blood pressure reduction did not vary by treatment [36]. Only the LCD group showed a substantial reduction in HbA1c after 6 months; however, HbA1c levels progressively reverted to baseline levels after 6 months. Moreover, the difference in HbA1c levels at 6 months in comparison with baseline did not reach statistical significance (*p* = 0.089). To minimize hypoglycemia, the oral medicine and insulin dose were reduced sequentially, and the insulin reduction was statistically significant only in LCD participants at 6 months. At 6 months, the variation in average insulin dose was marginally significant (*p* = 0.046) [30]. On 12-month follow-up, compared to baseline, all nutritional interventions resulted in positive effects regarding lipid profiles and blood pressure, insulin, and glucose concentrations. However, participants in the LCD group of healthy individuals showed an increase on LDL cholesterol concentrations.

According to the trial carried out by Gardner et al. [34], the alterations on LDL cholesterol levels that occurred during a year greatly favored a healthy dietary model that was low in fat. Concentrations of HDL cholesterol increased significantly, and triglyceride levels decreased considerably, in the group that followed the healthy low-carbohydrate low-fat diet when compared to the group that followed the healthy low-fat diet. There was no noticeable variation among the diet groupings concerning the rate at which the prevalence of metabolic syndrome decreased. 

In a study performed by Bazzano et al. [35], no significant difference between the two groups concerning serum concentrations of cholesterol and LDL cholesterol was noticed after a period of 12 months. The HDL cholesterol levels of the people in the LCD group increased significantly further than the HDL cholesterol levels of the people in the low-fat group (the median alteration at 12 months was 0.18 mmol/L [7.0 mg/dL] [CI, 0.08 to 0.28 mmol/L 3.0 to 11.0 mg/dL]; *p* = 0.001). The mean variation in alteration at the first year was 0.44 (CI, 0.71 to 0.16); *p* = 0.002; only the LCD individuals exhibited considerable reductions in HDL cholesterol fractions. The levels of serum triglycerides dropped significantly in both groups, with lower levels being observed in the LCD group (mean alteration at year 1 was 0.16 mmol/L [14.1 mg/dL] [CI, 0.31 to 0.01 mmol/L 27.4 to 0.8 mg/dL]; *p* = 0.038). This was ascribed to a reduction in the consumption of carbohydrates in both groups. In the study by Trico et al. [29], dietary treatments boosted both baseline and glucose-accelerated insulin production, as measured by C-peptide deconvolution. Concerning indicators produced by models, cell function and cell glucose sensitivity improved following both diets; however, incretin hormones decreased. Comparable or decreased peripheral insulin concentrations, in spite of increased insulin secretion, were ascribed to higher fasting and overall insulin clearance, which marginally increased at the LCD model compared to the Mediterranean diet model (fasting: +21.4 [81]% and +8.5 [37.7]%, respectively, *p* = 0.06; total: +21.4 [81]% and 3.0 [33.1]%, respectively, *p* = 0.06).

## 4. Discussion

The study by Jenkins et al. [32] indicated that a vegan LCD resulted in a moderately greater body mass decrease than that of an HCD (7% vs. 6% decreases, respectively) over a 6-month ad libitum period. These decreases were the same as those noted for low-carbohydrate ‘Atkins-like’ dietary models [37]. When compared with the HCD, LCD intervention that included vegetable proteins and oils resulted in considerably lower levels of LDL-C. The above was the case regardless of whether or not the diet was high in total carbohydrates. Other LCD studies have not shown significant LDL-C reductions [38], and LDL-C decrease was not supported by any previous LCD research. This is ascribed to the fact that the other trials on LCDs contained a significant amount of protein and fat that originated from animal sources. The prolonged decrease in LDL-C, which was related to a slight additive body mass lowering on the 6-month self-designated diet, is a component of nutrition that has the potential to be meaningful in reducing the risk of coronary heart disease over the long run [39]. Participants who followed the diet over a period of six months showed this drop in their body weight. An LCD, in which choices for vegetable fat and protein were promoted, revealed a higher reduction in the TC:HDL-C fraction compared to that observed at 6 months in body mass-lowering surveys employing either a Mediterranean Diet or an HCD [40]. This was found in the aforementioned study as well.

Even though the LCDs emphasized vegetable fat and protein sources, this remains the case. During the metabolic phase, those who followed an LCD exhibited total compliance to the main nutritional ingredients, which were 33.6% of what was supplied [41]. These essential elements of a healthy diet were nut products, vegetable proteins (such as soy and gluten), and viscous fibers. This level of compliance is comparable to the 43.3% that was observed concerning the nutritional selection in the analysis that compared the metabolism control between a 1-month study and an ad libitum 6-month study. The analysis compared the two studies to determine which one had a higher level of adherence [42]. In this specific survey, the reduction in LDL-C that occurred during the month of LCD was also higher compared to that which occurred over six months of eating, despite the fact that the treatment differences were comparable [43].

In the clinical trial by Gardner et al. [34], 609 healthy adults with overweight or obesity were enrolled. The participants of this study were free of diabetes and were randomly included in a healthy low-fat vs. a healthy LCD. In the first year, no considerable change in body mass was noted. Moreover, no substantial associations among nutrition and three SNP multilocus genotype patterns or nutrition and baseline insulin excretion on first-year body mass lowering were noted. These findings were noted concerning the same first-year body mass lowering in both nutritional intervention groupings, which was higher compared to 5% of baseline body mass. A substantial variety of body mass alteration, indicating about 40 kg within each nutritional intervention group (from lowering about 30 kg to increasing about 10 kg in participants’ classes, as assessed by a nutritional evaluation), was established by alterations in blood lipid indices and robust treatment reliability. A high number of participants, good retention rate, substantial weight loss, weight loss variability, and great compliance and nutrition diversity were reported. The survey was well arranged to identify considerable associations by the initial variables of concern if they occurred. Nevertheless, no such impacts were noted.

Changes in reduction among the two groups were not considerable and not significant at a clinical level. In the study by Trico et al. [29], including individuals with obesity and an-insulin-resistance at high risk to develop type 2 diabetes, it was demonstrated that a low-carbohydrate/high-protein diet may be a reasonable alternative to a Mediterranean diet with balanced macronutrient composition for body mass reduction and glucose control. The participants on LCD group showed higher definite and percentage body mass decreases when compared to the Mediterranean diet, despite the similar everyday caloric limitation. The two nutritional treatments seem to exhibit similar efficiency in improving insulin resistance and fasting hyperinsulinemia, which are crucial pathologic processes for developing diabetes [44], while improving β-cell function and endogenous insulin clearance. The individuals on the LCD group accomplished a ~60% higher body mass reduction compared to the Mediterranean diet, at a level (>5%) which seems effective in reducing diabetes prevalence and enhancing cardiovascular risk factors in patients affected by obesity [45].

In comparison with typical dietary interventions, a low-carbohydrate/high-protein diet exhibits better efficiency in decreasing body mass in a brief time period [46].This finding was ascribed to the satiating effects of protein and its effect on inhibiting hunger [47], and to the rise in thermogenesis due to food ingestion [48] compared to carbohydrates and fat. Particularly, in people affected by obesity eligible for bariatric surgery, an LCD is suggested as a reasonable way to achieve a 5–10% body mass reduction in the preoperative period, which facilitates surgery and decreases the probability of adverse events [42]. The greater effectiveness of LCDs on body mass reduction and its maintenance was not reliably noted in long-term trials, performed over a 6- to the 24-month period [48]. Thus, combined dietary studies have suggested LCDs implementation to achieve rapid body mass reduction, followed by Mediterranean-style dietary regimens for long-term weight maintenance [49].

Studies regarding LCDs have shown positive results in the short term. However, most studies failed to follow participants in the long term. Considering the fact that we need long-term results, adherence to a dietary pattern needs to be long-term as well. Acceptance, palatability, cost, and a healthy relationship with food—in contrast to disordered eating behaviours—are very important factors for long-term adherence to a dietary pattern and one’s health. High-quality, long-term studies on LCDs are still scarce [50,51]. Barber et al. emphasized the safety concerns for this dietary pattern, including potential nutritional deficiencies that can occur when restricting major and important food groups, the unknown long-term effects of ketosis on bone and kidney health, as well as the possibility of hyperuricemia [51,52]. Further mentioned implications are the financial cost for the people and the cost to the planet and climate change, and of course, the long-term effect on mental health as food is more than energy supply [51].

Schutz et al. also focused on the scarcity of evidence for the LCDs in contrast to the available evidence on other dietary patterns such as the Mediterranean Diet, and the potential medical risks regarding the nutritional adequacy, the kidney health, the risk of metabolic acidosis and the impact of a high-fat diet in people affected by obesity [53]. The effect of carbohydrate restriction on longevity has also been explored by Seidelmann et al., who found reductions in longevity after carbohydrate restriction, with consumption of protein and fat of animal origin [54].

Moreover, an important dietary concern regarding LCDs is their fiber content. Fiber intake is important for health, especially to gastrointestinal health and function [55], whereas low fiber diets are associated with metabolic and gastrointestinal diseases and functional disorders (such as constipation, diverticular disease, and colon cancer) as well as mental well-being via the gut–brain axis [56].

Regarding the long-term efficacy of LCDs on patients with type 2 diabetes, the existing evidence has suggested that after 12 months the efficacy of LCDs attenuated, and even patients’ quality of life, was lower, at a non-significant level though [57]. Additionally, regarding weight loss, long-term data suggest that LCDs were no better than other approaches [58], even when genotype patterns of insulin secretion were taken into account [33]. However, some metabolic effects could be favorable after 1 year of follow-up [59].

Furthermore, in the long term, in mice, LCDs induced non-alcoholic fatty liver disease and decreased insulin sensitivity, which should alarm the scientific community [60].

### Side Effects of Low Carbohydrate Diets

Low-carbohydrate dietary patterns are quite the opposite of the well-studied Mediterranean diet, and various concerns are raised regarding their safety and side effects.

Besides the benefits of LCDs regarding epilepsy, weight loss, and blood glucose control—at least in the short term—it is important to note that LCDs exhibit certain “side effects” and disadvantages. LCDs are comprised of a variety of dietary patterns such as protein and fat content. Moreover, source and quality can differ, hence their disadvantages, and safety may also differ from person to person [61,62,63]. Carbohydrates are the main source of energy for the body, hence, when the intake of carbohydrates has limited the use of lipids for energy can lead to the so-called “keto flu”, with adverse effects including headaches and nausea. If not addressed, the shortage of carbs will also result in hormonal shifts, fluid, and mineral loss, and fluid and mineral retention. Overall, effects such as constipation and gastrointestinal discomfort, fatigue, nausea, dizziness, cravings, and both hunger and decreased appetite have been documented; however, few studies have systematically examined these side effects [64].

Due to the fact that LCDs can differ, while in the real world the definition of carbohydrates may differ from the scientific definition, the side effects can be different. In addition to this, carbohydrates, and especially fiber, are essential for gut microbiota. Trials in human and animal models have mixed results, with some studies showing positive effects on bacteria and their metabolites’ functions, whereas others show negative effects with decreased diversity and dysbiosis [65]. Furthermore, the long-term effect of LCDs on health and the impact of ketosis on health are other important aspects that need to be thoroughly examined [51,53].

The risk of nutritional deficiencies is an important problem that may occur in a diet where a macronutrient is strictly avoided, while even with supplementation of essential micronutrients, the scientific community should not forget the synergistic effect of foods’ micronutrients and antioxidants [51]. Long-term adoption of a dietary pattern is important for its successful results, and the palatability and cost [66], as well as the freedom of being able to consume the foods one wants, maybe a few of the disadvantages of adhering to this dietary pattern.

## 5. Conclusions

For decades, an alternative proposal for a “low-carb” diet has been emerging, which, although initially severely criticized, has in recent times been shown to be just as effective, if not more so, compared to the low-fat diet approach for body mass lowering and for many associated metabolic diseases such as diabetes. The effectiveness of LCDs (which are also high in protein) focuses not so much on calories and limiting energy intake, but on macronutrients and their proportion in the diet, with an emphasis on low-carbohydrate food consumption. The most popular LCD is the Atkins diet with only 5–10% of energy (about 20 g per day) coming from carbohydrates and a high-fat content and intake. However, several notable studies have suggested that LCDs are efficient in body mass lowering, and are now a recommendation for certain medical conditions such as diabetes mellitus and fatty infiltration. LCDs may lead to an improvement in glycosylated hemoglobin (HbA1c). LCDs may also be efficient in improving reduction in people with type 2 diabetes in the short term; however, it remains unknown whether this approach is more successful in the long term than any other approach. Overall, future clinical studies are required for more precise conclusions to be taken concerning the health benefits of LCDs against obesity and diabetes.

## Figures and Tables

**Table 1:** Characteristics of the chosen studies.

Study Type	Measured Parameters	Number of Patients	Duration	Effects	References
Randomized clinical trial	Body weight, Insulin secretion, insulin clearance, β-cell function	36	4 weeks	The mean body mass lowering was 5%, being 58% higher in the LCD group compared to Mediterranean-group. Fasting plasma glucose and glucose resistance remained unaltered by the nutritional interventions. The two nutritional interventions showed the same efficiency in improving insulin tolerance and fasting hyperinsulinemia while increasing endogenous insulin clearance and β-cell glucose sensitivity.	Tricò et al. [29]
A prospective randomized parallel trial	Body weight, HbA1c, HDL, LDL, Insulin doses	61	2 years	At 2 years participants had an average weight loss of 5.1 kg. HbA1c fell in the LCD group only. At 6 months, HDL-cholesterol enhanced with the LCD, while LDL-cholesterol was not different between groups. Insulin doses were decreased in the LCD group.	Guldbrand et al. [30]
Randomized controlled trial	Body mass reduction, LDL-C, Triglyceride, Cholesterol, Apolipoprotein B:A1 ratios	39	6 months	LCD showed a decrease LDL-C and triglyceride compared to low-carbohydrate treatment	Jenkins et al. [32]
Randomized controlled trial	Age, Body weight, BMI, overall body fat mass, waist circumference, blood lipids, triglycerides, cholesterol, HDL, LDL, Adiponectin, Leptin	170	6 months	Subjects lost 7.3 ± 4.0 kg loss of weight with a reduced-carbohydrate diet and 6.2 ± 4.2 kg with a reduced-fat diet (*p* < 0.001) within each group. Calories restriction results in identical considerable reductions in left ventricular mass with high carbs diets (5.4 ± 5.4 g) or low-fat diets (5.2 ± 4.8 g; *p* < 0.001) within each group.	Haufe et al. [33]
Randomized clinical trial	Body weight, insulin secretion	609	12 months	Weight alteration at 1st year, was −6.0 kg for the healthy LCD. There was no considerable nutrition–genotype pattern interplay or nutrition–insulin secretion interplay within the 1st year of body mass lowering	Gardner et al. [34]
Randomized parallel-group trial	Body weight, fat mass, HDL, cholesterol, triglycerides	148	12 months	At 1st year, individuals on the LCD showed higher reductions in body mass, fat mass, a ratio of overall HDL cholesterol, and triglyceride concentrations and higher enhancement in HDL cholesterol concentrations compared to a low-fat diet.	Bazzano et al. [35]
Randomized clinical trial	BMI, body mass, fat mass, waist circumference, waist-to-hip ratio, systolic blood pressure, total cholesterol, LDL-cholesterol, insulin, Homeostatic Model Assessment for Insulin Resistance (HOMA)	331	9 months	The reduction at 9 months of BMI, weight, fat mass, systolic blood pressure, insulin levels and HOMA were higher in the diet with low carbs than the diet with no carbs. With both interventions, leptin concentration was reduced.	de Luis et al. [36]
